# Mercury Exposure through Fish Consumption in Traditional Communities in the Brazilian Northern Amazon

**DOI:** 10.3390/ijerph17155269

**Published:** 2020-07-22

**Authors:** Sandra de Souza Hacon, Marcelo Oliveira-da-Costa, Cecile de Souza Gama, Renata Ferreira, Paulo Cesar Basta, Ana Schramm, Decio Yokota

**Affiliations:** 1 Fundação Oswaldo Cruz, Escola Nacional de Saúde Pública Sérgio Arouca, Rio de Janeiro 21041-210, Brazil; pcbasta@ensp.fiocruz.br (P.C.B.); schrammana@posgrad.ensp.fiocruz.br (A.S.); 2 WWF-Brasil, Brasília 70377-540, Brazil; marcelo@wwf.org.br; 3 Instituto de Pesquisas Científicas e Tecnológicas do Amapá, Av. Feliciano Coelho, 1509. Trem, Amapá 68901-025, Brazil; cecile.gama@iepa.ap.gov.br; 4 Iepé-Instituto de Pesquisa e Formação Indígena, Macapá, Amapá 68908-120, Brazil; renata.ferreira@institutoiepe.org.br (R.F.); decio@institutoiepe.org.br (D.Y.)

**Keywords:** protected areas, traditional communities, mercury contamination, health risk assessment, Amazon

## Abstract

Artisanal small-scale gold mining (ASGM) is the main source of anthropogenic mercury emissions and contamination in Latin America. In the Brazilian northern Amazon, ASGM has contaminated the environment and people over the past century. The main contamination route is through fish consumption, which endangers the food security and livelihoods of traditional communities. Our study aims to assess the potential toxicological health risks caused by the consumption of Hg-contaminated fish across five regions in Amapá State. We sampled 428 fish from 18 sites across inland and coastal aquatic systems. We measured the total mercury content in fish samples, and the results were applied to a mercury exposure risk assessment targeting three distinct groups (adults, women of childbearing age, and children). Mercury contamination was found to exceed the World Health Organization’s safe limit in 28.7% of all fish samples, with higher prevalence in inland zones. Moreover, the local preference for carnivorous fish species presents a serious health risk, particularly for communities near inland rivers in the region. This is the first study to provide clear recommendations for reducing the mercury exposure through fish consumption in Amapá State. It builds scientific evidence that helps decision-makers to implement effective policies for protecting the health of riverine communities.

## 1. Introduction

Artisanal small-scale gold mining (ASGM) activities have significantly expanded in the Amazon over the last two decades. ASGM is a major cause of deforestation and habitat degradation in the Brazilian northern Amazon [1,2,3], particularly on the borders between Guyana, French Guiana, Suriname, and Venezuela, and the Brazilian states of Roraima, Amapá, and Pará (also known as the Guiana Shield Ecoregion) [4]. The ecoregion covers 250 million hectares and contains one of the largest complexes of uninterrupted primary tropical forests on Earth [5]; the region therefore plays a critical role in mitigating global climate change and preserving biodiversity and freshwater ecosystems.

The Brazilian state of Amapá borders French Guiana and Suriname, and almost 72% of its territory is formally protected. It consists of five indigenous territories, four quilombolas territories, and nineteen conservation units (twelve federal, five state, and two municipal), covering a total area of 115.656 km^2^. These areas are crucial for human livelihoods, especially for communities that rely on their natural resources. However, the protection of these areas has been hampered by weak governance, lack of financial resources for protective management, and different anthropogenic pressures. Among those threats, the widespread use of mercury (Hg) in ASGM has led to the contamination of both the environment and the regional population.

The ASGM sector is the leading source of mercury emissions, contamination, and consumption in Latin America and the Caribbean [6]; thus, the traditional communities that depend on natural resources for their survival are highly vulnerable to ASGM activities. This activity is generally coordinated by illegal networks and conducted by wildcat gold miners, and dealers [7]. ASGM has adversely affected the health of riverside communities—particularly indigenous people [8,9]—that rely on fish as their main protein source [10,11]. Fisheries in the Amazon provide food security and reduce malnutrition rates, as fish are the dominant dietary source of essential amino acids, lipids with fatty acids, minerals, and vitamins [12,13].

In ASGM-dominated regions, human exposure to Hg is mainly through bioaccumulation and biomagnification of methylmercury from fish consumption. In the Brazilian Amazon, indigenous people, riverside communities, fishermen, quilombolas, peasants, and extractivists inhabit the areas near rivers, bays, and streams and are therefore highly exposed to Hg compounds. This reality is aggravated by the social vulnerability of exposed communities, including reduced access to healthcare, formal education, regular income, basic sanitation, and potable water. In addition, these communities suffer from high rates of malnutrition, particularly in children under 5 years of age. They are also vulnerable to a high number of infectious and respiratory diseases (such as malaria, hantavirus pulmonary syndrome, leishmaniasis, yellow fever, etc.) that are exacerbated by both changes in land use and climate [14].

Therefore, mercury contamination in the Amazon is both a national and international concern. In this study, we aimed to develop a better understanding of the risks associated with fish consumption in various regions of the northern Amazon. Although a few studies have shown mercury contamination in fish in the region [5], this is the first to conduct an investigation on human Hg exposure in the study region. We assessed the potential toxicological health risks from mercury exposure through fish consumption for different age groups across five regions in Amapá State.

## 2. Methods

### 2.1. Study Area

**Figure 1:**
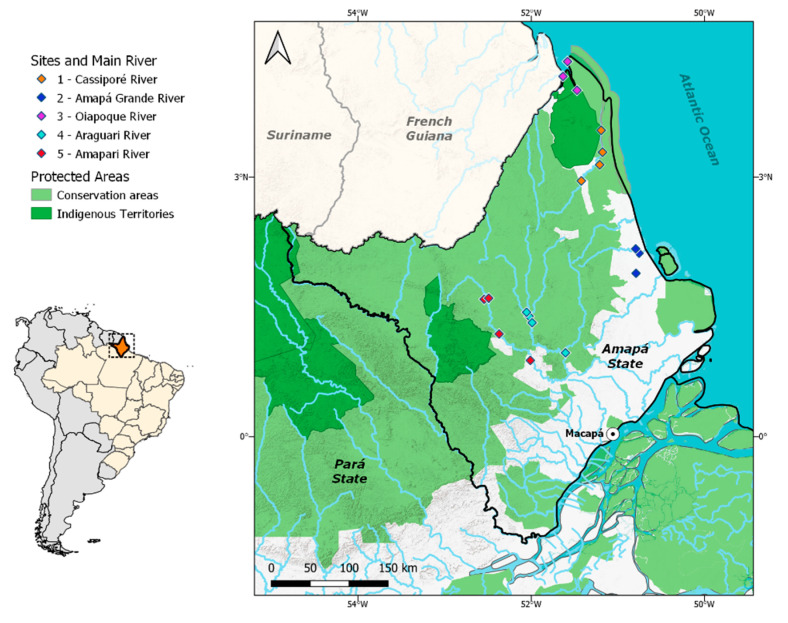
Map of the study area indicating the sampling sites, protected areas, and indigenous territories.

We assessed five regions in the Amapá State territory, including some of the most biodiverse and economically significant river basins in the region (Figure 1). Watersheds in the state play an important social, cultural, and economic role, and fish resources in the region are heavily influenced by the Amazon River and Atlantic Ocean. We chose rivers that bordered protected areas (Table 1) due to the higher occurrence of mineral deposits [15] and higher number of ASGM sites surrounding Amapá’s protected areas [16]. Sampling sites were set up at potential ASGM sites (active and inactive). These sites were identified based on gold mining deforestation maps [3], literature reviews [5,17], the presence of local communities, the influence of coastal tides or inland waters, sampling logistics, the distance to protected areas, and interviews with managers of protected areas and locals with knowledge of the regional gold mining history. Sampling sites 1, 2, and 3 are directly influenced by coastal tides and are therefore considered coastal zones, while sites 4 and 5 are located in inland waters and are therefore referred to as inland zones.

### 2.2. Fish Sampling and Preparation

Between August 2017 and May 2018, we collected tissue samples from 428 fish from 45 different species at 18 sites across the five regions (Table 1). The fish were caught by local fishermen hired to support the research project. According to the local fishermen, each fish species was captured using specific gear; therefore, gillnets, hooks and hand lines, fishing rods and long lines were used. Each specimen was identified to the species level, and a minimum of 70 g of muscle tissue (free of skin) was extracted from the dorsal region of the fish body. All dissection tools were sterilized before sampling to avoid contamination. Samples were stored in ice boxes and frozen for transportation to the laboratory. The study was conducted in accordance with the Brazilian regulation (IN 03/2014), under the license SISBIO 58296-1. 

### 2.3. Mercury Analysis

Total mercury (THg) was measured by cold vapor atomic fluorescence spectrometry at the Analytical Chemistry Laboratory of the Pontific University of Rio de Janeiro [23]. The analytical quality was determined by control strict blanks with duplicate analysis and compared to the analytical results of certified reference materials (DORM-2, Dogfish Muscle Certified Reference Material for trace elements, National Research Council, Ottawa, ON, Canada). Average recovery values were >96% of the certified value analytical results; and the relative standard deviation (RSD) was <15%. Total mercury analysis from the wet weight was adopted.

### 2.4. Data Analysis

The data are presented as mean ± standard error and percentiles. We applied the Kruskal–Wallis test to determine the mean differences in THg concentrations between sampling locations and trophic levels.

The risk assessment approach in this study was based on the method used by the US Government Agency for Toxic Substances and Diseases Registry [24]. The method includes four steps: (i) hazard identification, (ii) dose–response assessment, (iii) exposure assessment, and (iv) risk characterization. Most risk assessment studies use secondary data; however, our Hg exposure assessment used information on the most consumed fish species in the region [5] as well as informal interviews with local fishermen and individuals from indigenous and riverine communities.

### 2.5. Estimated Daily Intake

To assess the health risks of total mercury exposure, we incorporated two scenarios for toxicological risk: (i) the critical scenario and (ii) the current scenario. The critical scenario assumes that some of the local communities—including the indigenous communities—consume larger quantities of fish due to the lack of other protein sources. The current scenario represents the eating habits of most of the communities in the study area. The estimated exposure dose of fish consumers from both coastal and inland zones were divided into three groups: (i) adults of both genders (age: 17 to 75), (ii) women of childbearing age (age: 17 to 49), and (iii) children/juveniles (age: 5 to 16). We estimated their daily intake (EDI) to determine each group’s daily Hg consumption [25]. The average daily fish consumption was based on 16 studies (Table 2) that assessed the dietary patterns and fish consumption of Amazonian communities.

The potential exposure dose (µg/kg/d) was calculated according to Equation (1) [26]:

(1) $$D_{pot}=C\times \frac{IR\times EF\times ED}{BW}\times \frac{1}{AT}$$

where *D_pot_* is the potential mercury intake dose (µg/kg/day); *C* is the total mercury concentration of fish species (µg/g); *IR* is the fish intake rate (kg/meal); *EF* is the frequency of exposure (meals /day); *ED* is the exposure duration (day, week); *AT* corresponds to the time weighted (day) (time weighted considers the duration of exposure); and *BW* is the body weight (kg).

The risk ratio was calculated as the ratio between the potential intake dose and the reference dose using the risk quotient equation (Equation (2)):

QR = *D_pot_*/RfD (2)

where QR is the risk quotient; *D_pot_* is the potential mercury intake dose (µg/kg/day); and RfD is the reference dose (1.6 μg/kg/week or 0.23 μg/kg/day [25]) of the substance or chemical element (Hg) that can be consumed weekly over a lifetime without appreciable risk to health. A QR of <1 indicates a low likelihood of adverse health effects, while QR > 1 indicates the possibility of adverse non-carcinogenic effects.

### 2.6. Model Input Variables

The description of the input variables for the mercury exposure model for both the critical and current scenarios are shown in Table 3. We assumed a 50% consumption of fish species in age groups with larger average Hg concentrations (carnivorous and omnivorous) as well as the groups with the lowest Hg concentration (herbivorous and detritivores). The literature review showed a high frequency of fish consumption and a high variability in the quantity of meals, ranging from 1 to 20 meals per week [38,40]. We therefore set an average of 10 meals of fish consumed per week, varying from 160 to 430 g/day, based on the characteristics of each group. We estimated the fish daily intake values based on the literature review (Table 2). The average body weight of the residents in the communities was obtained from research on family budgets that presented the anthropometry and nutritional status of children, adolescents, and adults in the state of Amapá [19].

To quantify exposure, we calculated the mean Hg concentrations of carnivorous and omnivorous fish as well as the 95% confidence interval (CI) of each mean. We also conducted a literature review to determine the frequency of fish meal consumption and estimated potential doses by assigning the 95% upper limit of the mean fish consumption. It was assumed 100% of THg concentration was methylmercury, since methyl Hg constitutes up to 98% of the Hg concentration in fish samples in the Amazon basin. The body weight was the same for both scenarios, and the factor absorption of total Hg as methyl Hg by exposed residents was assumed to be 100% for both scenarios. We used the lognormal function to estimate the exposure dose.

## 3. Results

### 3.1. Mercury Levels in Fish Species and Trophic Levels

**Figure 2:**
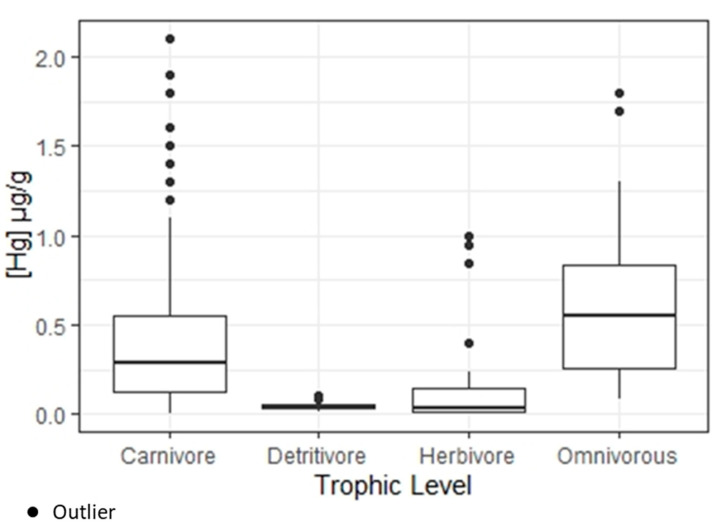
Distribution of mercury concentrations in fish (μg/g) by trophic level.

All of the 428 fish sampled in this study had detectable levels of mercury, and 28.7% exceeded the World Health Organization’s (WHO) mercury threshold (0.5 μg/g) for human consumption. The Hg concentration in fish exceeded the safe limit in 77.6% of carnivores, 20% of omnivorous, and 2.4% of herbivores. Carnivorous fish accounted for 76% (*n* = 325) of the total samples, with average Hg concentrations of 0.4 μg/g (SD = 0.38) and a concentration range of 0.008–2.1 μg/g. Interestingly, the Hg concentration in omnivorous fish averaged 0.60 μg/g (SD = 0.42) and reached a maximum of 1.8 μg/g. This could be explained by changes in fish feeding habits. For instance, fish from the omnivorous species *Pimelodus ornatus* can change their diet depending on the resources available in the environment. Two herbivorous species showed unexpectedly high Hg concentrations of 1.0 μg/g for *Mylesinus paraschombourgkii* (flaviano) and 0.85 μg/g for *Myloplus ternetzi* (pacu). We also observed significant differences in the average Hg concentrations between trophic levels (*p* < 0.001) (Figure 2).

**Figure 3:**
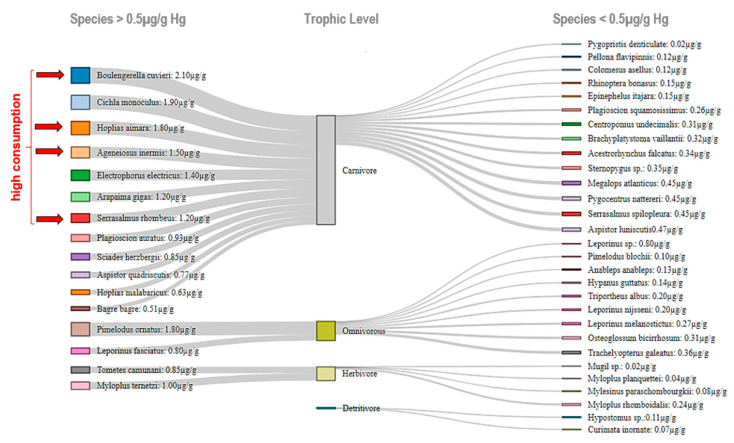
Sankey diagram categorizing the studied fish species and trophic levels by total mercury concentrations (≥0.5 μg/g and ≤0.5 μg/g).

Four of the seven species with the highest Hg concentrations (Figure 3) are among the most consumed species in the region [5]. The highest level (2.1 μg/g) was detected in *Boulengerella cuvieri* (pirapucu), followed by *Cichla monoculus* (tucunaré), and *Hoplias aimara* (traírão), which are all carnivorous species.

### 3.2. Mercury Levels at Sampling Sites (Inland and Coastal Zone)

**Figure 4:**
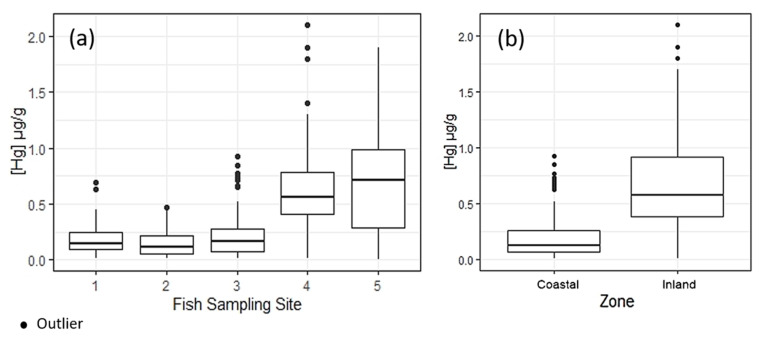
Distribution of mercury concentrations in fish (μg/g) in (**a**) the sampling sites and (**b**) the aggregated zones (Kruskal–Wallis: 95% CI, *p* < 0.001).

The median Hg contamination of inland fish samples was 0.580 µg/g for carnivores (*n* = 131), 0.045 µg/g for herbivores (*n* = 15), and 0.680 µg/g for omnivores (*n* = 33); samples of detritivorous species were not collected. In coastal zones, the median Hg concentrations were 0.165 µg/g for carnivores (*n* = 194), 0.019 µg/g for herbivores (*n* = 5), 0.290 µg/g for omnivores (*n* = 15), and 0.044 µg/g for detritivores (*n* = 35). The median Hg levels of carnivorous and omnivorous species showed no statistical differences in both aquatic systems. Hg contamination exceeded the WHO limit in 28.7% of all samples, in 5.2% of coastal samples, and 61.5% of inland samples (*p* < 0.001) (Figure 4). It is therefore 29 times more likely to observe Hg levels above the safe limit in fish samples from inland areas (95% CI, 15.3–54.6) relative to those in coastal zones. The regional variability of Hg levels in the study region is likely related to the different hydrological, physicochemical, and environmental dynamics between inland and coastal areas.

The highest Hg concentrations were detected in *Serrasalmus rhombeus* (piranha preta), *Pimelodus ornatus* (mandi casaca), and *Hoplias aimara* (traírão) in the inland water zone, and *Sciades herzbergii* (bagre guribu), *Megalops atlanticus* (pirapema), and *Hoplias malabaricus* (traíra) in the coastal zone.

### 3.3. Health Risk Assessment

Fish from the inland zone had higher levels of Hg compared to fish from the coastal zone (Figure 4b). This is reflected by the potential dose and toxicological QR of total mercury for both exposure scenarios in coastal and inland zones (Table 4). The potential THg dose of the three groups was mostly above the acceptable reference dose of 1.6 μg/kg/week or 0.23 μg/kg/day [25]. In the critical scenario in both zones, the potential THg dose showed high risks of adverse health effects in all groups, regardless of location. The exception was the current scenario for women of childbearing age in the coastal zone (0.08 μg/kg/day). Children/juveniles in the inland zone showed significantly greater risks (*p* ≤ 0.005) under the critical scenario (55) relative to the current scenario (37.5). Our results highlight the elevated health risks associated with fish consumption in the study area.

## 4. Discussion

High levels of mercury were detected in different fish species and trophic levels in remote and legally protected areas of the northern Amazon; this suggests a high risk of Hg contamination in local fish consumers. Based on the risk assessment of fish Hg concentrations from 18 sites in Amapá State we recommend a low-risk fish diet for the three different groups. We found that the local preference for carnivorous fish species presents a significant health risk for local communities, particularly in inland water zones. As top predators, carnivorous fish bioaccumulate larger quantities of Hg throughout their lifecycle.

The mercury dynamics in aquatic ecosystems are complex and influenced by many factors, including soil type, river flow, fish trophic level, age of fish, season, pH, the productivity of aquatic ecosystems, characteristics of methylation sites, and others [41,42]. As most of the fish samples (81%) were caught during the dry season (with only one field campaign during the rainy season), our study may be limited by the lack of seasonal data; however, we observed no significant difference in Hg concentration in the fish sampled in the dry and rainy seasons (*p* ≥ 0.05). Another potential source of bias is the lack of detailed primary data on local eating habits, including dietary patterns and meal composition, particularly with regard to the fish intake dose. To minimize this potential bias, we incorporated a conservative approach by assuming a low daily fish intake in relation to the current exposure scenario and in response to the high frequency and quantity of weekly meals [27,40]; this is due to the high regional heterogeneity in the Amazon region, where fish consumption is higher in some communities due to the lack of alternative protein sources.

Furthermore, erosion can significantly influence THg levels in Amazonian aquatic ecosystems by mobilizing inherited Hg in soils. Consequently, efforts to reduce Hg exposure—particularly in populations downstream of ASGM sites—must also address soil erosion, with a particular focus on vulnerable rivers and streams in riparian forests [43]. In addition, changes in land cover/use leads to frequent forest fires, which releases large quantities of Hg to the atmosphere [44] and aquatic systems [42]. These issues require the political will to implement effective policies to reduce the drivers of deforestation in the Amazon.

Comparing the current and critical scenarios in the coastal zone, we observed no significant difference in the potential doses of children/juveniles based on the acceptable methylmercury dose (1.6 μg/kg/week or 0.23 μg/kg/day) [25]. In contrast, a significant difference was observed in the potential doses of children in the inland zone (*p* ≤ 0.005). For this group, we assumed children consume the same fish species as adults but in smaller quantities. Furthermore, children are at significant risk of Hg exposure because of their higher Hg doses and lower body weights (approximately half that of adults). The potential dose for the other two groups (adults and pregnant women) in both zones was double the WHO’s daily recommended dose at 0.5 μg/kg/day. The potential dose for women of childbearing age in the coastal zone was within the safe limit at 0.08 μg/kg/day. Nevertheless, under the critical scenario in the coastal zone, the toxicological risks for adults (men and women) was 26 and 20 times higher than the daily reference dose for adults and women of childbearing age, respectively. Our results suggest that both groups in the coastal zone that consume carnivorous fish five times per week on average have significantly higher health risks from THg contamination, even in areas where lower fish Hg levels were observed.

Both scenarios showed a high risk of Hg contamination in the inland zone. The highest risks were observed in children at doses of 37.5 μg/kg/week and 55 μg/kg/week. The consumption of fish with high levels of Hg therefore poses significant health risks for this group. Adults under the current and critical scenarios in both zones had a potential dose of 1.9 μg/kg/day and 9.9 μg/kg/day, respectively, inferring a low risk for the current scenario and very high risk for the critical scenario. For pregnant women, the potential dose was 1.8 μg/kg/day under the current scenario, and the dose for the critical scenario was 4.3 times that of the current scenario. Many studies assessing riverbank populations in the Amazon show that a small proportion of the communities consume carnivorous fish at a frequency of 10 meals a week [15,27,36,39].

These communities have been exposed to Hg contamination for over a century through the ingestion of mercury contaminated fish. This long-term Hg exposure has led to potential adverse health effects, such as fetal impairment in women of childbearing age [45,46,47].

Our findings suggest that the consumption of the carnivorous fish species *Cichla monoculus, Hoplias malabaricus, Aspistor quadriscutis, Ageneiosus inermis, Plagioscion auratus,* and *Sciades herzbergii* should not exceed 200 g per week. This is equivalent to a dose of 1.4 μg/kg/week for a 70 kg adult. In contrast, we identified no health risks regarding the consumption of herbivorous (such as *Mugil* sp. (tainha)) and detritivorous species (such as the *Hypostomus* sp. (acari/cascudo) and *Curimata inornata* (branquinha)) in the coastal communities. Moreover, to minimize health risks, *Ageneiosus inermis* (mandubé), *Boulengerella cuvieri* (pirapucu), *Cichla monoculus* (tucunaré), and *Hoplias aimara* (traírão) should only be consumed once a month. Pregnant women should avoid consuming carnivorous fish and reduce the intake of omnivorous fish during the pregancy. 

## 5. Conclusions

To our knowledge, this is the first study to provide clear recommendations for reducing the Hg exposure through fish consumption in local communities in the northern Amazon. High levels of mercury were detected in different fish species in the study area, suggesting a high risk of Hg contamination in local fish consumers. The highest risk of Hg contamination was observed in children in the inland zone. To minimize health risks, we proposed a maximum weekly fish intake for different trophic levels based on both the toxic characteristics of Hg and human susceptibility. We suggest that the consumption of the carnivorous fish species should not exceed 200 g per week, with special attention to the consumption of mandubé, pirapucu, tucunaré and trairão, that should be consumed once a month.

The study locations were selected in protected and conserved areas, including Brazil’s largest natural reserve (Tumucumaque National Park). These regions provide ecosystem services for traditional communities and maintain their social and cultural values, including the incorporation of fish resources for human livelihoods. 

Due to the persistence of Hg in the environment, legal and illegal ASGM activities in Amapá continue to impact the health and livelihoods of traditional communities who are dependent on fish as a primary protein source. This situation has been exacerbated by the global increase in the price of gold, which has driven the expansion of illegal ASGM activities in the Amazon and further threatens the environment and the human rights of local communities. This also inhibits the ability of vulnerable populations to achieve their sustainable development goals.

In this context, it is critical to assess the impacts of Hg contamination on local communities and their environment. There is a strong need to build scientific evidence that helps to develop effective mitigation measures and policies to tackle these issues. It is crucial to assess the impact of mercury contamination on human health at regional scales. Policy makers must also devise and implement effective mitigation measures to guarantee the food security of indigenous communities; this includes the proposition of nutritional alternatives and the moderation of fish consumption without compromising cultural traditions.

## Figures and Tables

**Table 1:** Description of the sampling sites and the surrounding regions.

Sites and Main River	Tributaries-Sampling Sites	Coordinates of Sampling Sites	Characteristics of the Region	Correlation to Gold Mining Activity
1-Cassiporé River (CR)	Maruani River	03°32′27.71″ N 51°11′34.23″ W	Coastal zone. Muddy water. High fishing pressure. Influence of tides and pororoca. Protected Areas: Cabo Orange National Park and Amapá State Forest. Communities (pop): Oiapoque, 27.270 ^a^; Calçoene, 11.117 ^a^; Vila Velha, 2.723 ^b^; Cunani (Quilombo) 940 ^b^. Indigenous lands: Juminã, 61 ^c^; Uaçá, 4.462 ^c^; Galibi, 151 ^d^.	Gold mining activity for over 250 years. Influence of Garimpo do Lourenço, located at the headwaters of the Cassiporé River. On-going mining processes in the indigenous land of Uaçá for gold research and exploration.
	CR-Bridge	02°57′25.57″ N 51°25′18.10″ W		
	CR	03°17′16.87″ N 51°10′36.68″ N		
	CR-Vila Velha	03°08′34.16″ N 51°12′36.16″ W		
2-Amapá Grande River (AG)	AG	02°10′16.19″ N 50°47′39.41″ W	Coastal zone. Muddy river bottom. High fishing pressure. Influence of tide and pororoca. Protected Areas: Cariaú River reserve, Maracá-Jipioca Ecological Station. Communities (pop): Calçoene, 11.117 ^a^; Pracuúba, 5.120 ^a^; Amapá, 9.109 ^a^ Tartarugalzinho, 17.315 ^a^.	This region is influenced by Garimpo do Tartarugalzinho. At least five other areas in the region constitute environmental liabilities: Bananal, Buracão, Fofoca, Mandiocal and Mineiro.
	AG-Confluence	02°07′13.50″ N 50°45′17.52″ W		
	Flechal	01°53′19.07″ N 50°47′25.27″ W		
3-Oiapoque River (OR)	Uaçá River	04°0′11.66″ N 51°28′16.28″ W	Coastal zone. Muddy water. High fishing pressure. Large population in the surroundings. Borders French Guiana. Communities (pop): Oiapoque, 27.270 ^a^ Calçoene, 11.117 ^a^. Indigenous lands: Juminã, 121 ^c^; Uaçá: 4 462 ^c^; Galibi: 151 ^d^.	Displacement of illegal ASGM toward French Guiana from 2002. Influence from ASGM sites in the whole basin from both countries.
	OR	04°9′49.10″ N 51°37′59.97″ W		
	OR (Bay)	04°20′9.00″ N 51°34′50.20″ W		
4-Araguari River (AR)	Tajauí River	01°23′27.10″ N 52°01′28.02″ W	Inland zone. Sandy River bottom. Illegal fishing. Protected Areas: Tumucumaque National Park, Amapá National Forest. Communities (pop): Pedra Branca do Amapari, 16.502 ^a^; Porto Grande, 21.971 ^a^; Ferreira Gomes, 7.780 ^a^.	Influence of the Amapari, Lourenço, Vila Nova, Capivara, Mururé ASGM sites.
	Falsino River	00°58′09.70″ N 51°36′16.90″ W		
	Mureré River	01°26′16.75″ N 52°03′14.30″ W		
	Mutum River	01°19′02.84″ N 51°59′23.09″ W		
5-Amapari River(AMR)	AMR	01°35′16.86″ N 52°32′38.18″ W	Inland zone. Sandy River bottom. Protected Areas: Tumucumaque Mountains National Park, Amapá National Forest. Communities (pop): Pedra Branca do Amapari, 16.502 ^a^; Serra do Navio, 5.397 ^a^. Indigenous: Wajãpi, 1.200 ^d^.	Four legal active ASGM sites. Illegal gold mining sites within PAs and prospection projects. The Tucano’s Gold Mine (Pedra Branca do Amapari) is the second largest gold mine in Brazil.
	Anacuí River	01°36′04.52″ N 52°29′26.99″ W		
	AMR/Jurupá River	01°11′13.52″ N 52°22′14.66″ W		
	AMR (Porto Terezinha)	00°52′53.52″ N 52°00′36.84″ W		

^a^ [18]. ^b^ [19]. ^c^ [20]. ^d^ [21]. ^e^ [22].

**Table 2:** Summary of articles that quantified fish consumption in communities in the Brazilian Amazon.

Reference						
Reference	Local	Year	Population	*N*	Consumption (g/Day; Meals/Week; Daily Eat %)	
					Average (SD)	Min-Max
[27]	Monte Alegre/PA	1993–1995	Families	35	369	−
[28]	Rio Madeira/RO	1991 and 1993	All ages	607	243 (135)	100–300
[29]	Rio Cuiabá/MT	1995–1996	>12	153	110.4 (152.5)	20–372
[30]	Maroni River, Community Wayana/French Guyana	1997	<1–14 Adults (15–45)	109 118	195 ± 9 372 ± 116	
[31]	Rio Negro/AM	1998–1999	Woman (1545–)	31	170.5	23–293
[32]	Alta Floresta/MT	2000–2002	All ages	251	−	3–180
[33]	Indigenous/PA	−	Munduruku	249	30.0 (16.6)	−
			Kayabi	47	110.4 (60.6)	−
[34]	Rio Tapajós/PA	2003	Woman > 15	121	124 (65)	−
			Men > 15	135	189.7 (105.5)	−
[35]	Lago Puruzinho/PA	2005–2006	All ages	120	406 (204.1)	−
[36]	Madeira River, Curiã Lake, Porto Velho	2009–2011	Grouped Villages		Daily eat (%) 5.8%–57.7%	
[37]	Madeira River/RO	2015	All	51	34.29	7.1–330
			Young	23	21.4	7.1–180
			Woman	12	34.3	10–328.6
			Adults (1549–)	28	51.1	10–330
[38]	Community of Brasília Legal, Tapajós/PA	2004	Woman	46	4.9 Meals/week	−
			Men	22	6 Meals/week	−
[39]	Lower Amazon River Trombetas River Purus River	2006–2008	Families	85	416.39 (209.12)	−
				183	490 (240.69)	−
				54	469 (207.72)	−
[40]	Tapajós River–Community of Barreiras/PA		Men (015–) (1660–) (5060–) Woman (015–) (1660–) (5060–)	28 21 55 26 40 17	Meals/week 15 ± 0 20 ± 1 20 ± 0 Meals/week 14 ± 0 20 ± 0 20 ± 0	
[7]	Middle Madeira River/RO	2009–2010	Grouped Villages	132	440	270–460

**Table 3:** Exposure parameters used for the health risk assessment of residents in areas influenced by coastal tides (sites 1, 2, and 3) and inland zones (sites 4 and 5).

Exposure Parameters by Scenario		Child & Juvenile (5–16)		Women and Men Adults (17–75)		Women of Childbearing Age (16–49)	
		Coastal Tidal	Inland	Coastal Tidal	Inland	Coastal Tidal	Inland
Current	[Hg]-omnivorous and carnivorous–Average (SD) µg/g	0.50 (0.37)	0.66 (0.61)	0.50 (0.37)	0.66 (0.61)	0.50 (0.37)	0.66 (0.61)
	[Hg]-detritivore and herbivore– Average (SD) µg/g	0.17 (0.18)	0.17 (0.22)	0.17 (0.18)	0.17 (0.22)	0.17 (0.18)	0.17 (0.22)
	Average fish consumption (kg/d) adjusted for trophic level	0.160 (0.49)	0.160 (0.49)	0.300 (0.154)	0.300 (0.154)	0.255 (0.135)	0.255 (0.135)
Critical	[Hg] (omnivorous & carnivore) 95% µg/g	1.2 (0.37)	1.48 (0.64)	1.2 (0.37)	1.48 (0.64)	1.2 (0.37)	1.48 (0.64)
	Average fish consumption (kg/d) 95% carnivorous	0.192	0.192	0.430	0.430	0.300	0.300
Average body weight kg (SD)		25 (12.0)	25 (12.0)	65 (6.3)	65 (6.3)	58 (4.7)	58 (4.7)

SD—standard deviation.

**Table 4:** Potential dose (*D_pot_*) and toxicological risk quotient (QR) of total mercury for both exposure scenarios in coastal and inland zones.

Exposure Scenario	Pot. Dose Child & Juvenile (5–16)		Pot. Dose Women and Men Adults (17–75)		Pot. Dose Woman of Childbearing Age (16–49)	
	μg/kg/day	μg/kg/week	μg/kg/day	μg/kg/week	μg/kg/day	μg/kg/week
	$D_{pot}$/d	$D_{pot}$/w	$D_{pot}$/d	$D_{pot}$/w	$D_{pot}$/d	$D_{pot}$/w
**Coastal Zone**						
Current scenario	6.8	34	0.5	2.5	0.08	0.4
Critical scenario	6.9	34.5	6.0	30	4.7	23.5
**Inland Zone**						
Current scenario	7.5	37.5	1.9	9.5	1.8	9
Critical scenario	11	55	9.9	49.5	7.7	38.5

## References

[1] Álvarez-Berríos N.L., Aide T.M. (2015). Global demand for gold is another threat for tropical forests. Environ. Res. Lett..

[2] Legg E.D., Ouboter P.E., Wright M.A.P. (2015). Small-Scale gold mining related to mercury contamination in the Guianas: A review. Prepared for WWF Guianas.

[3] Rahm M., Jullian B., Lauger A., de Carvalho R., Vale L., Totaram J., Cort K.A., Djojodikromo M., Hardjoprajitno M., Neri S. (2015). Monitoring the Impact of Gold Mining on the Forest Cover and Freshwater in the Guiana Shield.

[4] Dolisca F. (2015). Final Evaluation. Guiana Shield Facility Project.

[5] Venturieri R., Oliveira-Da-Costa M., Gama C., Jaster C.B. (2017). mercury contamination within protected areas in the brazilian northern amazon-amapa state. Am. J. Environ. Sci..

[6] UNEP (2014). The Minamata Convention on Mercury and Its Implementation in the Latin America and Caribbean Region. http://mercuryconvention.org/Portals/11/documents/publications/report_Minamata_LAC_EN_FINAL.pdf.

[7] Doria C.R.D.C., Machado L.F., de Souza S.T.B., Lima M.A.L. (2016). Fishing in riverside communities in the region of the middle Madeira River, Rondônia. Novos Cad. NAEA.

[8] Barbosa A.C., Silva S.R.L., Dórea J.G. (1998). Concentration of mercury in hair of indigenous mothers and infants from the Amazon basin. Arch. Environ. Contam. Toxicol..

[9] Vega C.M., Orellana J.D.Y., Oliveira M.W., Hacon S.S., Basta P.C. (2018). Human mercury exposure in yanomami indigenous villages from the brazilian amazon. Int. J. Environ. Res. Public Health.

[10] Martín-Doimeadios R.R., Nevado J.B., Bernardo F.G., Jiménez-Moreno M., Arrifano G.P.F., Herculano A.M., Nascimento J.L.M., Crespo-Lopez M.E. (2014). Comparative study of mercury speciation in commercial fishes of the Brazilian Amazon. Environ. Sci. Pollut. Res..

[11] Santos E.C.O., Jesus I.M., Brabo E.S., Loureiro E.C.B., Mascarenhas A.F.S., Weirich J., Câmara V.M., Cleary D., Deoliveirasantos E., Britoloureiro E. (2000). Mercury exposures in riverside Amazon communities in Pará, Brazil. Environ. Res..

[12] Licona S.P.V., Negrete J.L.M. (2019). Mercurio, metilmercurio y otros metales pesados en peces de Colombia: Riesgo por ingesta. Acta Biológica Colombiana.

[13] Organização das Nações Unidas para Agricultura e Alimentação (FAO) (2015). The State of Food and Nutritional Security in Brazil.

[14] Ellwanger J.H., Kulmann-Leal B., Kaminski V.L., Valverde-Villegas J.M., Da Veiga A.B.G., Spilki F.R., Fearnside P.M., Caesar L., Giatti L.L., Wallau G.L. (2020). Beyond diversity loss and climate change: Impacts of Amazon deforestation on infectious diseases and public health. Anais Academia Brasileira Ciências.

[15] Oliveira M.J. (2010). Diagnosis of the Mineral Sector of the State of Amapá.

[16] Instituto Socioambiental-ISA (2019). Indigenous Peoples in Brazil. General Framework of Peoples. https://pib.socioambiental.org/pt/Quadro_Geral_dos_Povos.

[17] Tritsch I., Marmoex C., Davy D., Thibaut B., Gond V. (2014). Towards a revival of indigenous mobility in French Guiana? Contemporary transformations of the Wayãpi and Teko Territories. Bull. Lat. Am. Res..

[18] Instituto Brasileiro de Geografia e Estatística (IBGE) Population Estimates. Rio de Janeiro. https://cidades.ibge.gov.br/.

[19] Instituto Brasileiro de Geografia e Estatística (IBGE) (2010). Family Budget Survey 2008–2009: Anthropometry and Nutritional Status of Children, Adolescents and Adults in Brazil.

[20] Instituto Brasileiro de Geografia e Estatística (IBGE) (2011). Ministry of Planning, Budget and Management. Synopsis of the Demographic Census 2010.

[21] Amapá Institute for the Environment and Spatial Planning – IMAP, Indigenous Lands of Amapá Amapá State Government. http://www.imap.ap.gov.br/conteudo/gestao/areas-indigenas.

[22] APINA, AWATAC, IEPÉ (2017). Socioenvironmental Management Plan Wajãpi Indigenous Land.

[23] U.S. Environmental Protection (2002). Agency EPA-method 1631, Revision E-Mercury in Water by Oxidation, Purge and Trap and Cold Vapor Atomic Fluorescence Spectrometry.

[24] Agency for Toxic Substances and Disease Registry (ATSDR) (1999). Toxicological Profile for Mercury.

[25] FAO/WHO (2008). Expert Committee on Food Additives. Sixty-first Meeting. Summary and Conclusions.

[26] USEPA (2002). Water Quality for the Protection of Human Health: Methylmercury.

[27] Cerdeira R.G.P., Ruffino M.L., Isaac V.J. (1997). Consumo de pescado e outros alimentos pela população ribeirinha do lago grande de Monte Alegre, PA. Brazil. Acta Amaz..

[28] Boischio A.A.P., Henshel D. (2000). Fish consumption, fish lore, and mercury pollution—Risk communication for the Madeira River people. Environ. Res..

[29] Yokoo E., Valente J., Sichieri R., Silva E. (2001). validation and calibration of mercury intake through self-referred fish consumption in riverine populations in Pantanal Mato-grossense, Brazil. Environ. Res..

[30] Fréry N., Maury-Brachet R., Maillot E., Deheeger M., De Mérona B., Boudou A. (2001). Gold-mining activities and mercury contamination of native amerindian communities in French Guiana: Key role of fish in dietary uptake. Environ. Health Perspect..

[31] Dórea J.G., Barbosa A., Ferrari I., De Souza J.R. (2003). Mercury in hair and in fish consumed by Riparian women of the Rio Negro, Amazon, Brazil. Int. J. Environ. Health Res..

[32] Hacon S., Rochedo E.R., Campos R.R., De Lacerda L.D. (1997). Mercury exposure through fish consumption in the urban area of Alta Floresta in the Amazon Basin. J. Geochem. Explor..

[33] Dórea J.G., De Souza J.R., Rodrigues P., Ferrari I., Barbosa A.C. (2005). Hair mercury (signature of fish consumption) and cardiovascular risk in Munduruku and Kayabi Indians of Amazonia. Environ. Res..

[34] Passos C.J.S., Silva D.S., Lemire M., Fillion M., Guimarães J.R.D., Lucotte M., Mergler D. (2008). Daily mercury intake in fish-eating populations in the Brazilian Amazon. J. Expo. Sci. Environ. Epidemiol..

[35] Oliveira R.C., Dórea J.G., Bernardi J.V.E., Bastos W.R., Almeida R., Manzatto A.G. (2010). Fish consumption by traditional subsistence villagers of the Rio Madeira (Amazon): Impact on hair mercury. Ann. Hum. Biol..

[36] Hacon S.S., Dórea J.G., Fonseca M.F., Oliveira B.A., Mourão D.S., Ruiz C.M.V., Gonçalves R.A., Mariani C.F., Bastos W.R. (2014). The influence of changes in lifestyle and mercury exposure in riverine populations of the Madeira River (Amazon Basin) near a hydroelectric project. Int. J. Environ. Res. Public Health.

[37] Mourão D.S. (2016). Evaluation of Exposure to Mercury in Riverside Communities in Porto Velho. Master’s Thesis.

[38] Mertens F., Fillion M., Saint-Charles J., Mongeau P., Távora R., Passos C.J., Mergler D. (2015). The role of strong-tie social networks in mediating food security of fish resources by a traditional riverine community in the Brazilian Amazon. Ecol. Soc..

[39] Isaac V.J., Almeida M.C. (2011). Fish Consumption in the Brazilian Amazon.

[40] Faial K., Deus R., Deus S., Neves R., Jesus I., Santos E., Alves C.N., Brasil D. (2015). Mercury levels assessment in hair of riverside inhabitants of the Tapajós River, Pará State, Amazon, Brazil: Fish consumption as a possible route of exposure. J. Trace Elem. Med. Biol..

[41] Gomes D.F., Moreira R.A., Sanches N.A.O., Vale C.A.D., Daam M.A., Gorni G.R., Bastos W.R. (2020). Dynamics of (total and methyl) mercury in sediment, fish, and crocodiles in an Amazonian Lake and risk assessment of fish consumption to the local population. Environ. Monit. Assess..

[42] Wasserman J.C., Hacon S., Wasserman M.A. (2003). Biogeochemistry of Mercury in the Amazonian Environment. Ambio J. Hum. Environ..

[43] Miserendino R.A., Guimarães J.R.D., Schudel G., Ghosh S., Godoy J.M., Silbergeld E.K., Lees P.S.J., Bergquist B.A. (2017). Mercury pollution in Amapá, Brazil: Mercury amalgamation in artisanal and small-scale gold mining or land-cover and land-use changes?. ACS Earth Space Chem..

[44] Hacon S., Artaxo P., Gerab F., Yamasoe M.A., Campos R.C., Conti L.F., De Lacerda L.D. (1995). Atmospheric mercury and trace elements in the region of Alta Floresta in the Amazon Basin. Water Air Soil Pollut..

[45] Davidson P.W., Myers G.J., Cox C., Axtell C., Shamlaye C., Sloane-Reeves J., Cernichiari E., Needham L., Choi A., Wang Y. (1998). Effects of prenatal and postnatal methyl mercury exposure from fish consumption on neurodevelopment: Outcomes at 66 months of age in the Seychelles Child Development Study. JAMA.

[46] Stern A.H., E Smith A. (2003). An assessment of the cord blood:maternal blood methylmercury ratio: Implications for risk assessment. Environ. Health Perspect..

[47] Oken E., Wright R.O., Kleinman K., Bellinger D., Amarasiriwardena C.J., Hu H., Rich-Edwards J.W., Gillman M.W. (2005). Maternal Fish Consumption, Hair Mercury, and Infant Cognition in a U.S. Cohort. Environ. Health Perspect..

